# Acute and Second‐Meal Effects of Oat Products on Postprandial Glucose Responses in Healthy Japanese Adults: A Randomized Crossover Pilot Study

**DOI:** 10.1002/fsn3.71791

**Published:** 2026-05-24

**Authors:** Hiroyuki Sasaki, Yuma Matsumoto, Hirofumi Masutomi, Katsuyuki Ishihara, Kazuko Hirao, Shigenobu Shibata, Akiko Furutani

**Affiliations:** ^1^ Research & Development Division Calbee, Inc. Utsunomiya Japan; ^2^ Division of Home Economics Aikoku Gakuen Junior College Tokyo Japan; ^3^ Graduate School of Biomedical and Health Sciences Hiroshima University Hiroshima Japan; ^4^ School of Advanced Science and Engineering Waseda University Tokyo Japan

**Keywords:** glycemic index, granola, oats, second‐meal effect

## Abstract

Oats are rich sources of soluble dietary fibers, particularly β‐glucan, which is linked to improved postprandial glycemic control. Oats and oat‐based cereals cause a slow and moderate increase in the postprandial blood glucose levels; however, the mechanisms by which different processing and preparation methods affect the glucose levels remain unclear. Consumption of fiber‐rich meals attenuates the glucose responses to subsequent meals, a phenomenon known as the second‐meal effect. This study examined the effects of various oat‐based foods with different processing and preparation conditions on the postprandial blood glucose and insulin responses and their second‐meal effect on the postprandial interstitial glucose in healthy Japanese adults. Two randomized crossover trials were conducted. In Experiment 1, 11 participants consumed white rice or one of three oat‐based foods (fruit granola, baked oatmeal, and cooked oats). Postprandial blood glucose and insulin levels were measured over 120 min. In Experiment 2, 10 participants underwent continuous interstitial glucose monitoring. They consumed white rice, oat‐based food, or no breakfast as their first meal, followed by a standardized second meal. In Experiment 1, all oat‐based foods in this study reduced the postprandial blood glucose levels compared to white rice. Fruit granola and baked oatmeal exhibited low incremental areas under the glucose curve and were classified as low glycemic index foods, whereas cooked oats exhibited a medium glycemic index. In Experiment 2, only cooked oats suppressed the interstitial glucose elevation after the second meal. Overall, these findings suggest that oat‐based foods may help moderate postprandial glycemia, and that cooked oats may show the second‐meal effect in healthy Japanese adults.

## Graphical abstract

Compared with white rice, oat‐based breakfasts reduced postprandial glycemia in healthy Japanese adults, with fruit granola and baked oatmeal classified as low‐GI foods and cooked oats as a medium‐GI food. Fruit granola and baked oatmeal elicited higher early insulin responses, whereas cooked oats showed a lower early insulin response. Cooked oats also improved the second‐meal glycemic response.

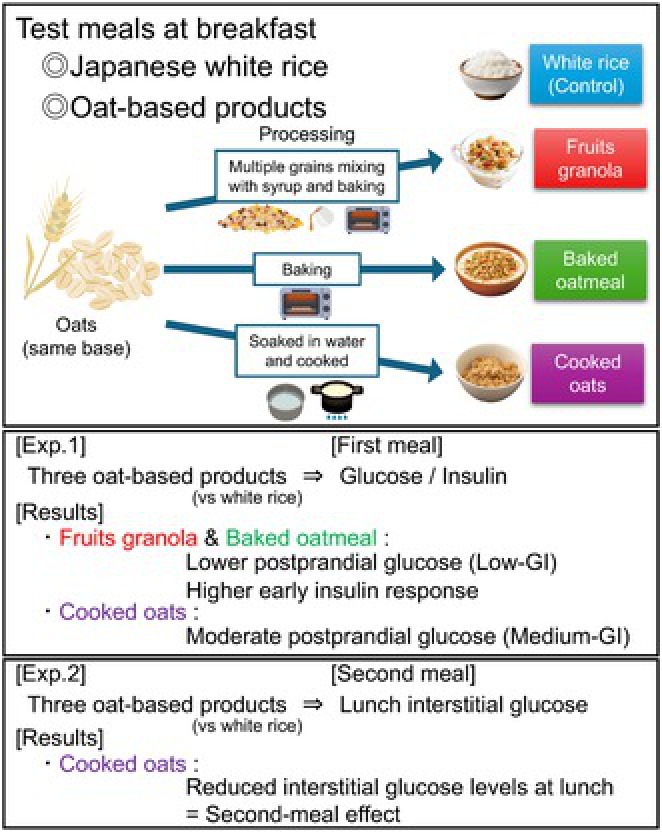

## 1 Introduction

Diabetes mellitus is a major public health concern in Japan. According to the International Diabetes Federation Diabetes Atlas, the number of adults aged 20–79 years with diabetes worldwide is projected to reach approximately 853 million by 2050. Additionally, the prevalence of prediabetes is notably high in Japan, with over 20 million individuals potentially affected by diabetes or related conditions (Federation 2025; Ikeda et al. 2017). Type 2 diabetes accounts for most cases, with increased urbanization and lifestyle changes serving as key contributors to its increasing global prevalence (Ogurtsova et al. 2017). Major risk factors for diabetes include sharp blood glucose spikes after meals and prolonged postprandial hyperglycemia (DECODE Study Group and The European Diabetes Epidemiology Group 2001; Group, D. S. 1999; Nakagami 2004). Furthermore, postprandial hyperglycemia induces inflammation in the vascular endothelial cells and promotes atherosclerosis development (Ansar et al. 2011; Mah and Bruno 2012). Therefore, dietary strategies to regulate blood glucose levels are crucial for effective health maintenance.

Glycemic index (GI) is a key indicator used to assess the impact of food on the blood glucose levels. First introduced by Jenkins et al. (1981), GI quantifies the postprandial blood glucose responses to specific foods (Jenkins et al. 1981). Based on their GI values, foods are commonly categorized into three groups: Low (≤ 55), medium (56–69), and high (≥ 70) GI groups (International Standards Organisation 2010). These classifications provide a practical reference point to the consumers for effective dietary decisions. Low‐GI foods lead to a slower increase in the blood glucose levels, facilitating a more sustained energy release and potentially reducing the sudden spikes in insulin secretion. Such foods are often high in dietary fibers (Wolever et al. 1994). In contrast, high‐GI foods cause a rapid and pronounced increase in the blood glucose levels, typically followed by a surge in the insulin levels. Frequent consumption of high‐GI foods is associated with the increased risk of insulin resistance and type 2 diabetes (Dickinson and Brand‐Miller 2005; Pi‐Sunyer 2002).

Another key concept of blood glucose regulation is the second‐meal effect. First introduced by Jenkins et al. (1982), this concept refers to the influence of the first meal on the postprandial blood glucose responses to a subsequent meal (second meal) (Jenkins et al. 1982). Notably, low‐GI food consumption enhances the second‐meal effect (Liljeberg and Björck 2000; Nilsson et al. 2008; Wolever et al. 1988). Water‐soluble dietary fiber intake is also associated with this effect (Brighenti et al. 2006; Jenkins et al. 1980; Rahat‐Rozenbloom et al. 2017). A recent human crossover study reported that the consumption of Jerusalem artichoke powder rich in water‐soluble dietary fibers reduces the postprandial blood glucose levels after lunch and dinner (Kim, Chijiki, et al. 2020). Furthermore, consumption of fiber‐rich snacks between lunch and dinner lowers the blood glucose levels after dinner and breakfast the next day (Kim, Nanba, et al. 2020; Masutomi et al. 2023).

Increasing dietary fiber intake is an important strategy to reduce the risk of postprandial hyperglycemia. Interestingly, average daily intake of dietary fibers among Japanese individuals remains below the government‐recommended target levels (21 g for males and 18 g for females) (Ministry of Health, L. a. W. J 2024). Dietary fibers are abundant in plant‐based foods, such as whole grains, vegetables, and seaweeds. Recently, oats have gained attention in Japan as rich dietary fiber sources, with various oat‐based products currently available in the market. Oats of genus *Avena* in the *Poaceae* family (Kamal et al. 2022) are commonly consumed as breakfast cereal, such as granola and oatmeal (Lin et al. 2025; Williams 2014). They contain approximately 9.4 g of dietary fiber per 100 g, over 30% of which is water‐soluble, with over 70% of the fraction being composed of beta‐glucan (Aoe 2016). Consumption of 1.14–5.3 g of oat‐derived β‐glucan reduces the peak postprandial blood glucose levels (Hlebowicz et al. 2008; Holm et al. 1992; Wolever et al. 2020; Zaremba et al. 2018). Similarly, intake of 1.7–6.0 g of β‐glucan lowers the incremental area under the blood glucose curve (IAUC) (Rieder et al. 2019; Steinert et al. 2016; Wolever et al. 2019; Zhu et al. 2020). β‐glucan exerts the second‐meal effect (Hossain et al. 2025). Characteristic water‐soluble dietary fibers, such as β‐glucan, attenuate the postprandial glycemic responses and contribute to the overall metabolic health.

Oats are processed and prepared in various ways, and they are most commonly consumed as cereals, such as oatmeal and granola. Recently, alternative methods, such as the preparation of rice‐like oats by adding water and upon cooking, have emerged. Although oat and oat‐based cereals lower the postprandial blood glucose levels, the mechanisms by which different processing and cooking methods affect these glycemic responses remain unclear (Wolever et al. 2019). Specifically, no study has evaluated the GI or second‐meal effect of differently processed oat products on glycemic responses in Japanese adults.

In this study, we conducted randomized crossover trials with healthy Japanese adults to assess the effects of oat‐based foods prepared using different processing and cooking techniques on the postprandial blood glucose levels, insulin responses, and second‐meal effects.

## 2 Materials and Methods

### 2.1 Participation Criteria

Healthy adults were recruited as study participants. Individuals were excluded from the study if they met any of the following criteria: (1) Allergy to test food, (2) history of glucose intolerance or abnormal results in a health checkup within the past 2 years, (3) fasting blood glucose level > 110 mg/dL, (4) body mass index (BMI) ≥ 30, (5) history of serious medical conditions, including liver, kidney, heart, lung, psychiatric, and hematologic diseases, (6) current use of medications, such as antihypertensive drugs, (7) pregnancy, lactation, or intention to become pregnant during the trial period, and (8) any other condition deemed inappropriate by the study supervisor. The study adhered to the ethical principles outlined in the Declaration of Helsinki and was approved by the Ethics Review Committee of Aikoku Gakuen Junior College. Written informed consent was obtained from all participants before study enrollment. The trial was registered on the University Hospital Medical Information Network (UMIN Clinical Trials Registry ID: UMIN000048192).

#### 2.1.1 Experimental 1: Postprandial Blood Glucose and Insulin Assessment

##### 2.1.1.1 Participants

Eighteen men and women aged 21–50 years consented to participate in the study. However, five individuals were excluded based on the eligibility criteria, resulting in 13 participants being formally enrolled in the study. These 13 participants were randomly divided into three groups (A, B, and C), with each group receiving the test meals in a different order.

**FIGURE 1:**
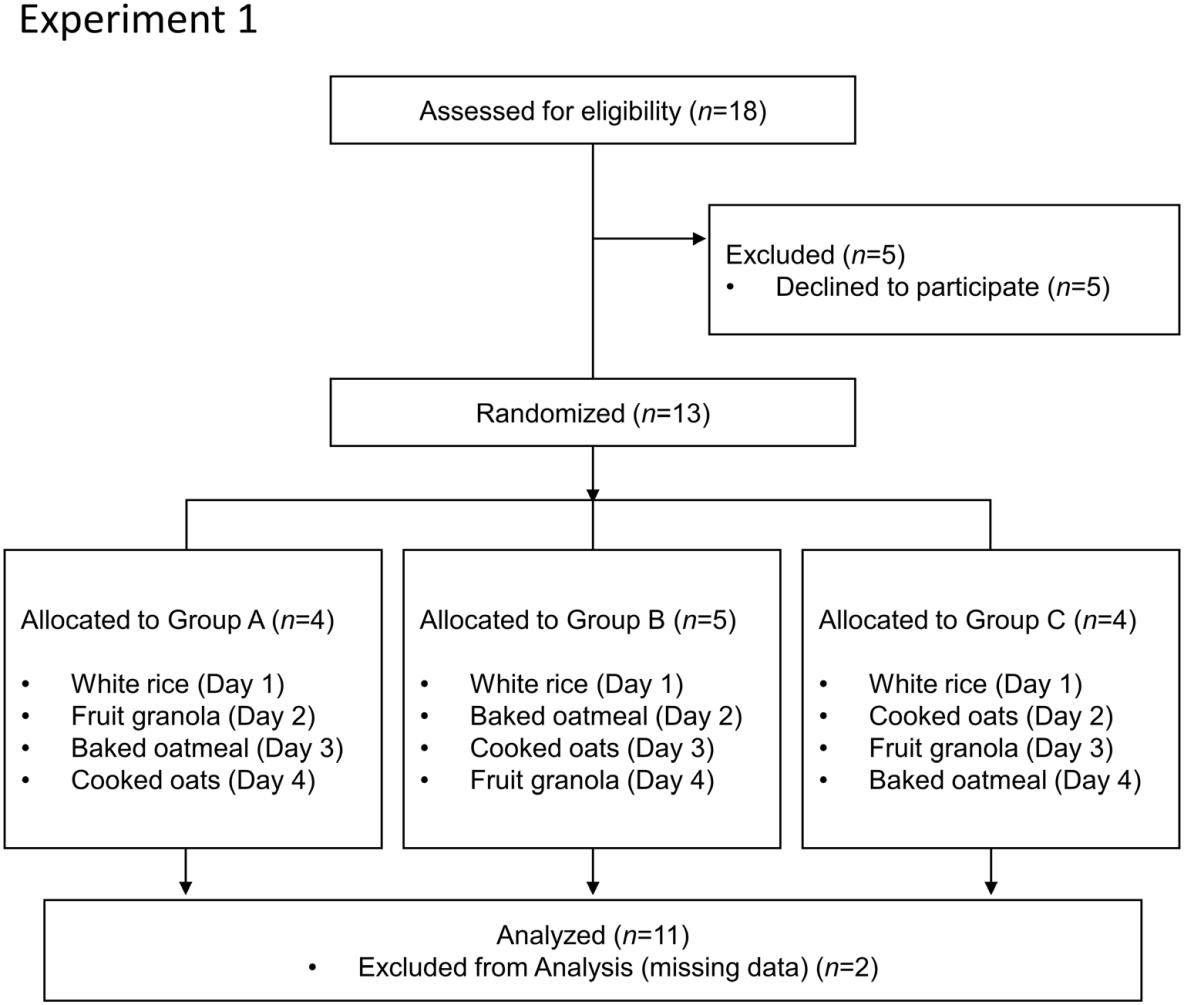
Flow diagram of Experiment 1 (postprandial blood glucose and insulin assessment). Eighteen individuals provided informed consent to participate in the study. Five participants were excluded from the study based on the eligibility criteria. The remaining 13 participants were randomly assigned to three groups (A–C) and completed the test meal regimen. Two participants were excluded from the final analysis because of missing data, resulting in only 11 participants (six males and five females) in the final analysis.

After completing all the scheduled interventions, two participants were excluded from the analysis because of missing data. Consequently, only 11 participants (six males and five females) were included in the final analysis (Figure 1). Age and BMI values of the participants are presented in Table 1.

**TABLE 1:** Participant age and body mass index (BMI) in Experiment 1.

	Male (*n* = 6)	Female (*n* = 5)	All (*n* = 11)
Age (years)	34.5 ± 3.2	41.4 ± 3.2	37.6 ± 2.4
BMI (kg/m^2^)	20.0 ± 1.0	23.0 ± 1.0	21.4 ± 0.7

*Note:* Values are expressed as the mean ± standard error of the mean (SEM).

##### 2.1.1.2 Study Design

Participants were instructed to fast from 10:00 p.m. in the evening before each test session, during which only water intake was permitted. They were also asked to maintain their usual sleep–wake schedule, to refrain from vigorous exercise, caffeine, and alcohol on the day before testing, and to commute to the study site in the morning as they normally would start behavior. Fasting blood samples were collected between 8:00 and 9:00 a.m., after which the participants consumed either a standard meal (white rice) or one of the three test meals. Because multiple participants were tested on the same morning and the test meals required different preparation procedures (e.g., reheating packed white rice), a 1 h time window was used to allow sequential preparation of meals and blood sampling while keeping the timing of measurements as consistent as possible. During the test period, the participants were allowed to drink up to 500 mL of water. They were instructed not to consume a large volume at once but to drink small amounts intermittently as needed. The exact timing and volume of water intake were not otherwise restricted or recorded.

Postprandial blood samples were collected from the forearm 15, 30, 45, 60, 90, and 120 min after meal consumption. Blood glucose levels were measured using an enzymatic method (CHOLESTECH LDX ANALYZER; Abbott Japan LLC, Tokyo, Japan), and insulin levels were determined using the Mercodia Human Insulin ELISA Kit (Mercodia, Uppsala, Sweden).

The test schedule was as follows: Standard meal was consumed first, followed by the test meals, in the order assigned to each group. Each test session was conducted with a one‐ or two‐day interval between trials. As the standard and test meals were visually distinguishable, the study was conducted using an open‐label design.

##### 2.1.1.3 Standard and Test Meals

Standard meal consisted of 150 g of packaged white rice (Sato no Gohan; Sato Food Industry Co. Ltd., Niigata, Japan), which is prepared from 100% Koshihikari rice. Standard meal was reheated immediately before serving in a microwave oven according to the manufacturer's instructions. The three test meals were fruit granola (Frugra; Calbee Inc., Tokyo, Japan), baked oatmeal (Baked Oats; Calbee Inc., Tokyo, Japan), and cooked oats (Gohan na Oats mugi; Calbee Inc., Tokyo, Japan).

General nutritional composition and dietary fiber content of each meal were analyzed by the Japan Food Research Laboratories. Based on these data, portion sizes were adjusted such that each standard or test meal provided 50 g of available carbohydrates. Other macronutrients and protein sources were not matched across test meals because the primary aim of this study was to compare commercially available oat‐based breakfast products as they are typically consumed in real life rather than to isolate the effects of individual macronutrients. In Experiment 1, fruit granola and baked oatmeal were served together with milk, whereas cooked oats and white rice were provided without milk or additional side dishes. Energy and nutrient compositions of each meal are listed in Table 2. White rice was used as the reference food for the calculation of glycemic index (GI) values (GI of white rice = 100), following methods previously applied in Japanese GI studies (Matsuoka et al. 2023; Sugiyama et al. 2003). Sugiyama et al. reported a strong correlation (*r* = 0.853) between the iAUC for white rice and that for a glucose solution, supporting the suitability of white rice as a reference food for GI determination in this context (Sugiyama et al. 2003).

**TABLE 2:** Energy and nutritional components of each test meal.

Nutritional components	White rice	Fruit granola + milk	Baked oatmeal + milk	Cooked oats
Energy (kcal)	234.0	415.1	432.5	345.0
Weight (g)	150.0	63.5 + 200	66.4 + 200	224.0
Carbohydrate (g)	50.0	50.0	50.0	50.0
Fat (g)	0.5	17.3	18.6	9.6
Protein (g)	3.8	12.0	15.2	10.1
Total dietary fiber (g)	2.3	5.7	6.3	9.2
Soluble dietary fiber (g)	1.2	3.5	3.7	5.4
Insoluble dietary fiber (g)	1.1	2.2	2.6	3.8
β‐glucan (g)	0.0	0.9	2.0	3.4

*Note:* Each test meal was adjusted to contain 50 g of the available carbohydrates. Weight values are shown in grams (g), and energy and nutrients are expressed per meal. Fruit granola and baked oatmeal were served with 200 mL of milk.

Fruit granola was prepared by steaming and flattening multiple grains, including oats, rye flour, and brown rice flour. These grain ingredients were milled, mixed with syrup, sheeted into a flat dough, baked in an oven, crushed, and then blended with dried fruits. Baked oatmeal was produced by steaming and flattening the oats only. The oat‐based ingredients were milled, mixed with syrup, sheeted into a flat dough, baked in an oven, and crushed. Cooked oats were provided as a retort pouch product developed by Calbee Inc. Oat groats were soaked in water, cooked, and then retort processed and packed at the manufacturing plant. All cooked oats used in this trial were reheated immediately before serving according to the manufacturer's instructions, without any additional ingredients. The ingredient lists of the tested meals are provided in Table S1.

##### 2.1.1.4 Analyses

Postprandial blood glucose and insulin level changes were calculated using the fasting values as the baseline. The maximum observed values were defined as peak changes. IAUC was calculated based on these changes, and GI was determined using the following formula:

$$\text{Glycemic index}=\frac{{\text{IAUC}}_{\text{test meal}}}{{\text{IAUC}}_{\text{standard meal}}}\times 100$$

Statistical analyses were conducted using GraphPad Prism (version 9.5.1; GraphPad Software Inc., Boston, MA, USA). All outcome variables were treated as continuous variables. Shapiro–Wilk test was used to assess the normality of each dataset.

Normality was not confirmed for the time‐course data of blood glucose and serum insulin levels measured at each time point. Therefore, the Friedman test was applied, followed by Dunn's multiple‐comparison test versus the standard meal (white rice), as the primary interest was to compare each test meal with the control condition. Dunn's procedure in GraphPad Prism provides multiplicity‐adjusted *p* values for comparisons with the control.

IAUC and peak blood glucose values passed the normality test and were analyzed via one‐way repeated‐measures analysis of variance, followed by Dunnett's post hoc test versus the standard meal (white rice). Dunnett's procedure also controls for multiple comparisons versus a single control. Statistical significance was set at *p* < 0.05.

#### 2.1.2 Experimental 2: Second‐Meal Effect Test

##### 2.1.2.1 Participants

Eleven participants (six males and five females) consented to participate in Experiment 2 and were enrolled in the study. They were randomly assigned to four groups (I, II, III, and IV), with each group receiving the test meals in a different order.

**FIGURE 2:**
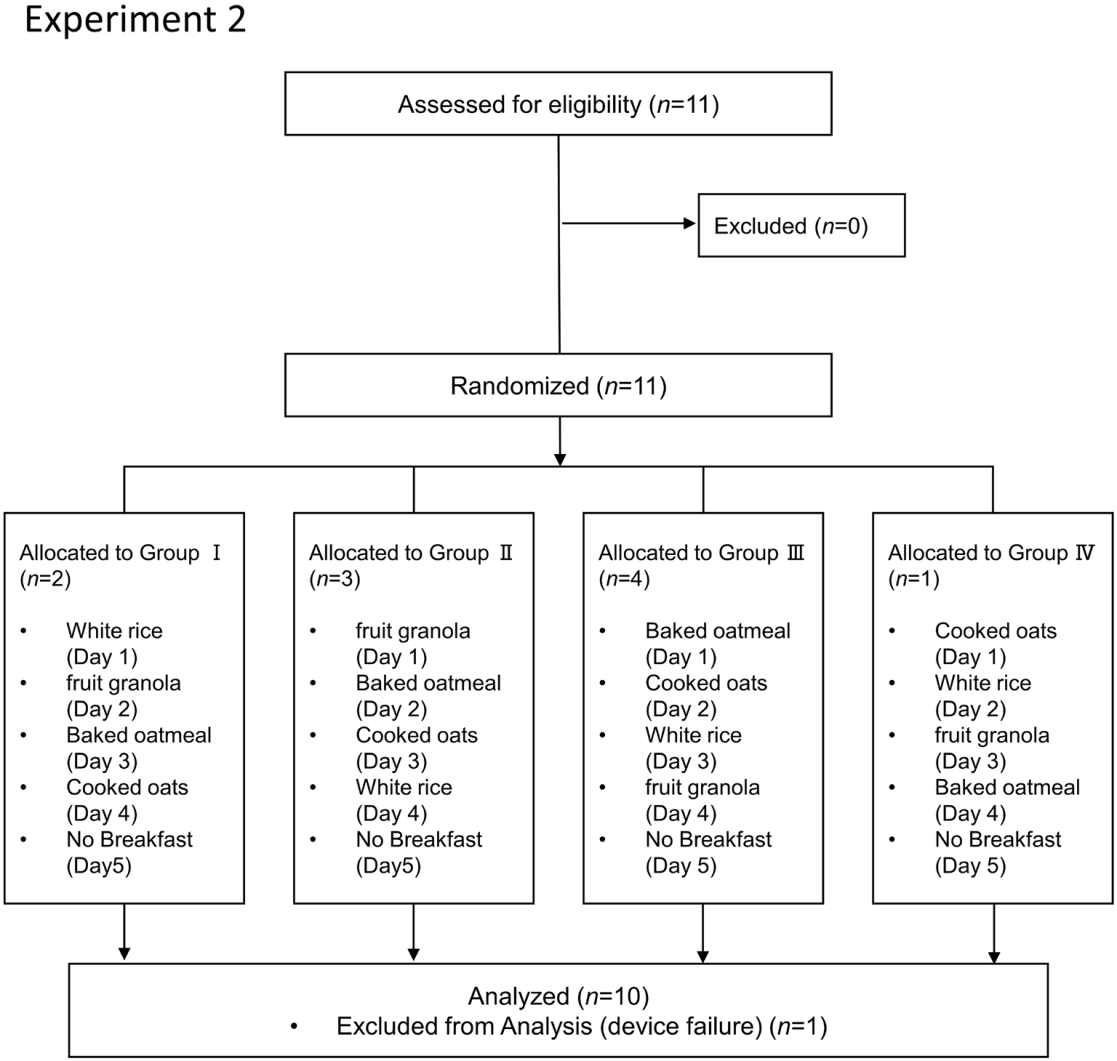
Flow diagram of Experiment 2 (second‐meal effect test). Eleven individuals (six males and five females) who provided informed consent to participate in Experiment 2 were enrolled in the study. The participants were randomly assigned to four groups (I–IV) and completed the test meal regimen in different orders. One participant was excluded from the final analysis because of missing data due to equipment failure. Consequently, 10 participants (six men and four women) were included in the final analysis.

After completing all the scheduled test sessions, one participant was excluded from the analysis because of missing data due to equipment failure. Consequently, 10 participants (six males and four females) were included in the final analysis (Figure 2). Age and BMI of the participants are presented in Table 3.

**TABLE 3:** Participant age and BMI in Experiment 2.

	Male (*n* = 6)	Female (*n* = 4)	All (*n* = 10)
Age (years)	32.7 ± 2.8	42.8 ± 2.8	36.7 ± 2.5
BMI (kg/m^2^)	20.1 ± 0.6	21.7 ± 1.6	20.7 ± 0.7

*Note:* Values are expressed by the mean ± SEM.

##### 2.1.2.2 Study Design

In Experiment 2, interstitial glucose levels were monitored using a continuous glucose monitoring system (FreeStyle Libre Pro; Abbott Japan LLC). FreeStyle Libre Pro measures the interstitial glucose levels every 15 min for up to 14 consecutive days. A previous study revealed the strong correlation between the interstitial and capillary blood glucose levels measured from the fingertips (Kumagai et al. 2019). FreeStyle Libre Pro‐determined glucose levels exhibit a high correlation with the capillary blood glucose levels, with a correlation coefficient of 0.95 within the 23–498 mg/dL range (Bailey et al. 2015).

Similar to that in Experiment 1, the participants were instructed to fast from 10:00 p.m. in the evening before each test day, with only water consumption permitted. Between 8:00 and 9:00 a.m., the participants consumed either a standard (white rice) or test meal as their first meal, followed by a second meal after 4 h. During the testing period, the participants were allowed to consume up to 500 mL of water. They were instructed not to consume a large volume at once but to drink small amounts intermittently as needed. The exact timing and volume of water intake were not otherwise restricted or recorded.

Each test session was conducted with a one‐ or two‐day interval between trials. As the standard and test meals were visually distinguishable, the study was conducted using an open‐label design.

##### 2.1.2.3 Standard and Test Meals

###### 2.1.2.3.1 First Meal

Standard meal consisted of 150 g of packaged white rice (Sato no Gohan; Sato Food Industry Co. Ltd.), which is prepared from 100% Koshihikari rice. Standard meal was reheated immediately before serving in a microwave oven according to the manufacturer's instructions. The test meals included fruit granola (Frugra; Calbee Inc., Tokyo, Japan), baked oatmeal (Baked Oats; Calbee Inc., Tokyo, Japan), and cooked oats (Gohan na Oats mugi; Calbee Inc., Tokyo, Japan). Additionally, a test condition was included in which the first meal was skipped, simulating a no‐breakfast scenario. As in Experiment 1, portion sizes of the cereal components were adjusted to provide 50 g of available carbohydrates for each first‐meal condition, and fruit granola and baked oatmeal were consumed with milk, whereas cooked oats and white rice were consumed without milk. The cooked oats used in Experiment 2 were identical to the prototype retort product used in Experiment 1.

###### 2.1.2.3.2 Second Meal

Second meal consisted of only 150 g of packaged white rice (Sato no Gohan; Sato Food Industry Co. Ltd.). A simple carbohydrate‐based meal was deliberately chosen as the standardized second meal to facilitate the detection of second‐meal effects of the oat‐based breakfasts and to minimize potential confounding by other nutrients, such as dietary fiber, fat, or protein in the second meal itself (e.g., fiber‐induced delayed gastric emptying (Jenkins et al. 1982)). In addition, a carbohydrate‐biased standardized meal design has been used in Japanese intervention studies to ensure a robust postprandial glucose excursion, for instance, selection of a standardized meal with a high carbohydrate ratio of 50% to induce meal‐related glucose elevations (Kuwahara et al. 2020). This design is consistent with previous second‐meal‐effect studies that also used white rice alone as the subsequent meal (Matsuoka et al. 2020; Taga et al. 2025).

##### 2.1.2.4 Analyses

Changes in interstitial glucose levels after the first and second meals were assessed using the start of each meal as the baseline. The highest value observed after each meal was defined as the peak interstitial glucose level. IAUC after the second meal was calculated based on these changes.

Statistical analyses were conducted using GraphPad Prism (version 9.5.1; GraphPad Software Inc.). Shapiro–Wilk test was used to assess the normality of the interstitial glucose changes. Normality was not confirmed for the glucose values at any time point. Therefore, a Friedman test was applied, followed by Dunn's multiple‐comparison test versus the standard meal (white rice) to evaluate the postprandial differences. As in Experiment 1, Dunn's procedure in GraphPad Prism provides multiplicity‐adjusted *p* values for these comparisons.

IAUC and peak interstitial glucose levels after the second meal met the assumptions of normality and were analyzed via one‐way repeated‐measures analysis of variance, followed by Dunnett's post hoc test versus the standard meal (white rice). Statistical significance was set at *p* < 0.05.

### 2.2 Sample Size Justification

The sample size for the present crossover experiments was estimated a priori using G*Power 3.1 (Heinrich‐Heine‐Universität Düsseldorf, Germany) for a one‐way repeated‐measures ANOVA. Based on previous intervention studies reporting reductions in postprandial glycemia with oat‐based products compared with control foods (Hlebowicz et al. 2008; Holm et al. 1992; Wolever et al. 2020), we assumed a large effect size (*f* = 0.40), with a two‐sided α level of 0.05 and a statistical power of 0.80. Under these assumptions, the required sample size was calculated to be 19 participants. Taking into account feasibility constraints and the burden associated with multiple test sessions, we initially aimed to recruit approximately 18 healthy adults for the study. However, the final analyzed sample sizes were 11 participants in Experiment 1 and 10 participants in Experiment 2, as an undesirable exclusion. Accordingly, the present findings should be interpreted with caution and confirmed in larger cohorts.

## 3 Results

### 3.1 Experiment 1

**FIGURE 3:**
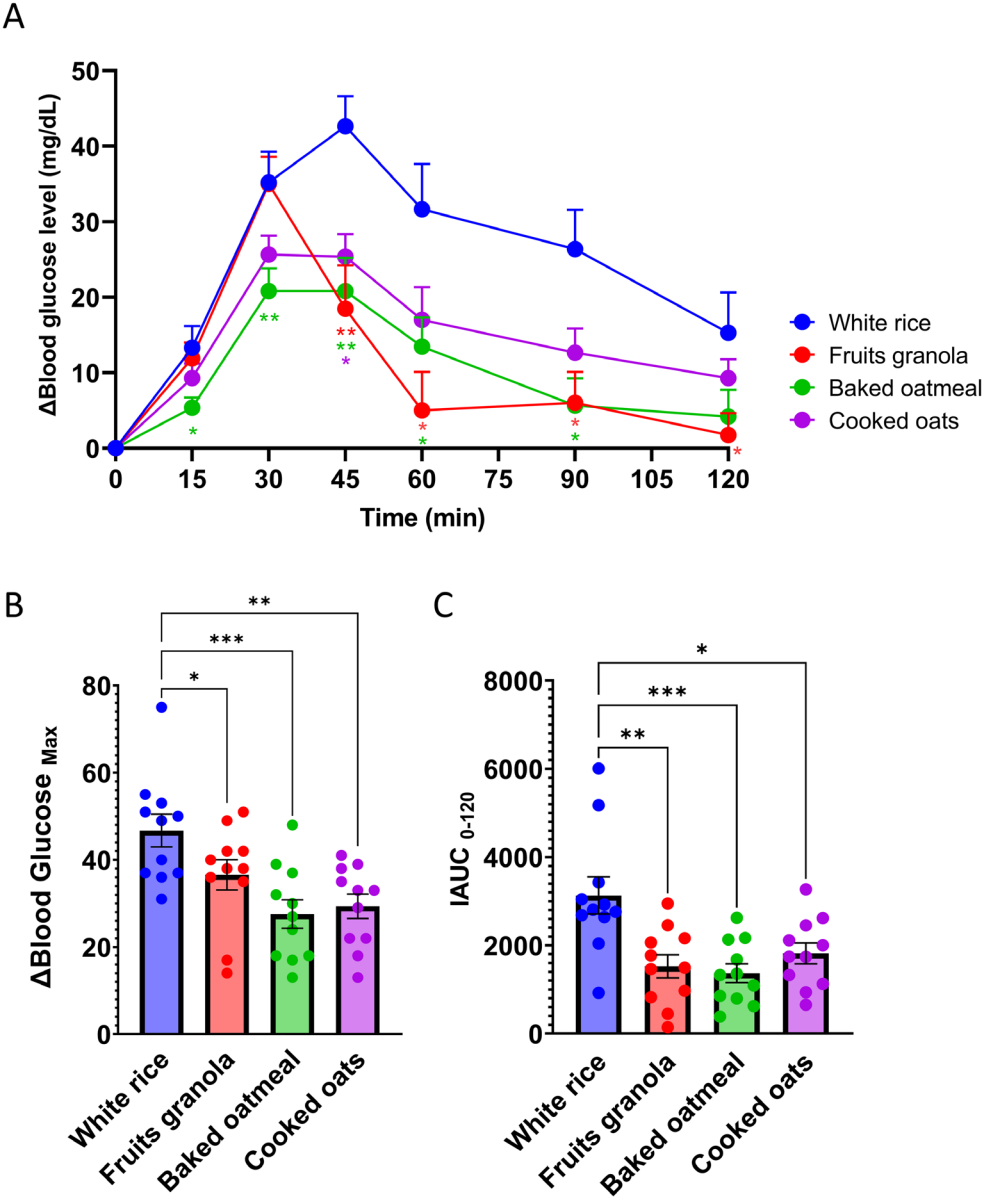
Oat‐based processed foods suppress the postprandial blood glucose spikes. (A) Postprandial blood glucose level fluctuations following the consumption of white rice, fruit granola, baked oatmeal, and cooked oats. (B) Maximum change in the blood glucose levels. (C) Incremental area under the blood glucose curve (IAUC). Values are expressed as the mean ± standard error of the mean (SEM; *n* = 11). *p < 0.05, **p < 0.01, and ***p < 0.001. Statistical significance in (A) was assessed using the Friedman test, followed by Dunn's multiple‐comparison test versus white rice. Statistical analyses in (B) and (C) were conducted via one‐way repeated‐measures analysis of variance (ANOVA) followed by Dunnett's post hoc test versus white rice.

Postprandial blood glucose levels after the consumption of white rice, fruit granola, baked oatmeal, and cooked oats are shown in Figure 3A and Table S2. Notably, meals containing oats (fruit granola, baked oatmeal, and cooked oats) exhibit smaller blood glucose fluctuations than white rice.

After fruit granola intake, blood glucose levels increased similarly to those in white rice for the first 30 min but began to decline after 45 min, remaining lower thereafter. In contrast, baked oatmeal and cooked oats caused a moderate increase in blood glucose levels immediately after consumption. Notably, baked oatmeal induced a smaller increase in blood glucose levels than white rice, as early as 15 min post‐consumption.

These differences were further reflected in the maximum changes in blood glucose levels: White rice showed an average increase in blood glucose levels of 45 mg/dL compared to the 36 in fruit granola, 27 in baked oatmeal, and 29 in cooked oats, which were lower than the blood glucose levels in white rice (Figure 3B; Table S3). Similarly, compared to white rice, all oat‐based meals reduced the IAUC by 12%–15% (Figure 3C; Table S3).

**FIGURE 4:**
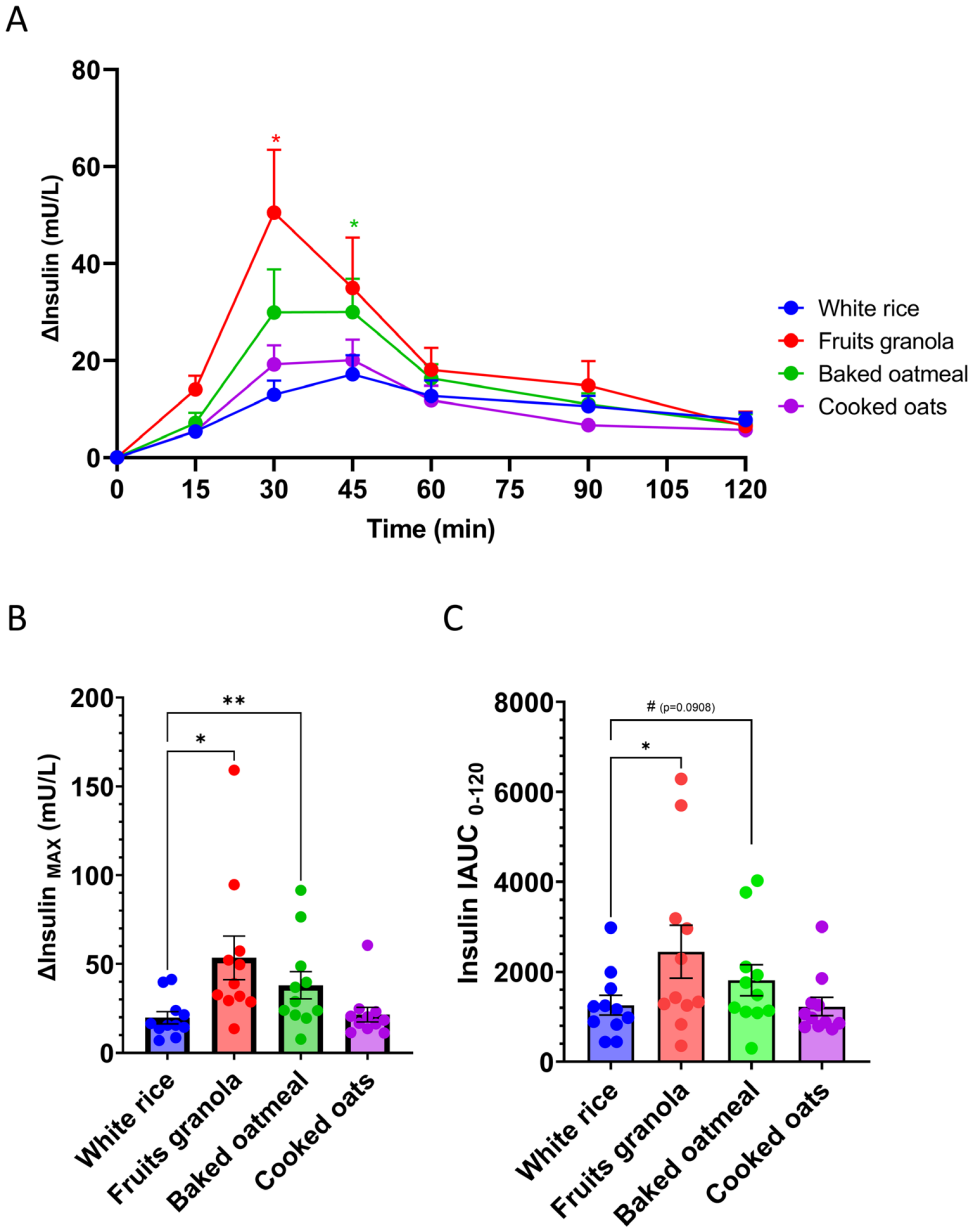
Fruit granola and baked oatmeal increase the postprandial insulin levels. (A) Postprandial insulin concentration fluctuations after the consumption of white rice, fruit granola, baked oatmeal, and cooked oats. (B) Maximum change in the insulin levels. (C) Incremental area under the insulin concentration curve (IAUC). Values are expressed as the mean ± SEM (*n* = 11). *p < 0.05 and **p < 0.01. Statistical significance in (A) was assessed using the Friedman test, followed by Dunn's multiple‐comparison test versus white rice. Statistical analyses in (B) and (C) were conducted via one‐way repeated‐measures ANOVA followed by Dunnett's post hoc test versus white rice.

Postprandial blood insulin level changes are shown in Figure 4A and Table S4. Upon white rice consumption, insulin levels peaked at 45 min, with an average increase of approximately 20 mU/L (Figure 4B; Table S4). Notably, no differences in the dynamic changes and peak levels of insulin were observed between cooked oats and white rice.

Fruit granola and baked oatmeal elicited different responses; insulin levels increased between 30 and 45 min post‐consumption, with fruit granola increasing the insulin levels at 30 min and baked oatmeal at 45 min compared to white rice (Figure 4A). Maximum increase in insulin concentration was also higher for fruit granola (53.5 mU/L) and baked oatmeal (38.1 mU/L) than for white rice (Figure 4B; Table S3).

IAUC of insulin was higher for fruit granola, whereas baked oatmeal exhibited a trend toward higher IAUC values than white rice (Figure 4C; Table S3).

Subsequently, GI values were calculated using white rice (GI = 100) as a reference. The resulting average GI values were 50.7, 44.4, and 65.8 for fruit granola, baked oatmeal, and cooked oats, respectively (Table 4). All test meals exhibited lower GI values than white rice. Based on the standard classification criteria, fruit granola and baked oatmeal were categorized as low‐GI foods, whereas cooked oats fell in the medium‐GI range.

**TABLE 4:** Glycemic index values for each test meal.

	White rice	Fruit granola	Baked oatmeal	Cooked oats
IAUC	3130.7 ± 419.1	1521.4 ± 261.0	1312.5 ± 231.4	1816.9 ± 236.2
Glycemic index	100.0 ± 0.0	50.7 ± 7.74	44.4 ± 6.86	65.8 ± 10.47

*Note:* Incremental area under the blood glucose response curve (IAUC) (mg*min/dL) was calculated for each test meal. Glycemic index (GI) was determined by expressing the IAUC as a percentage of the IAUC for white rice, which served as the reference meal (GI = 100). Values are expressed as the mean ± SEM.

### 3.2 Experiment 2

**FIGURE 5:**
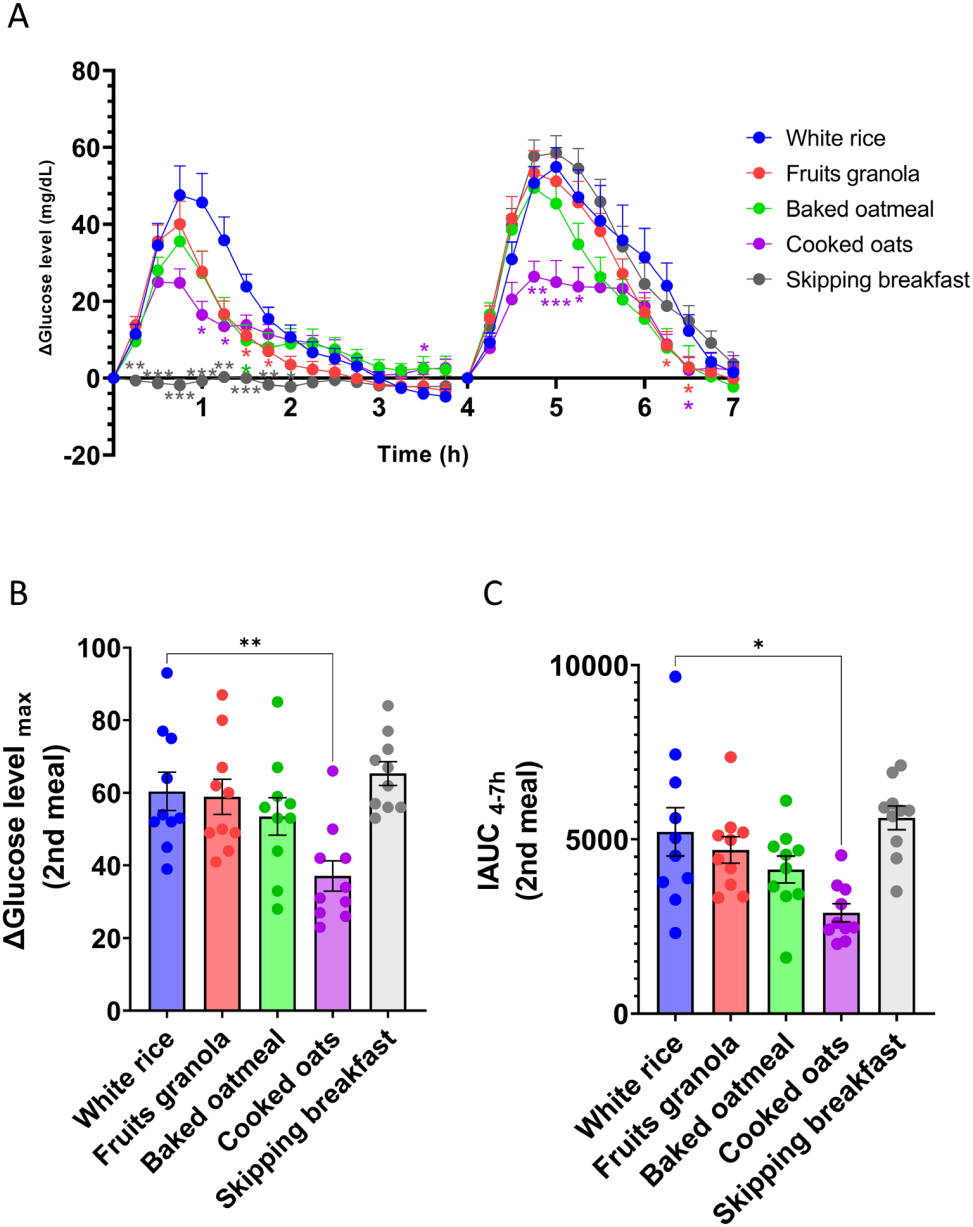
Consuming cooked oats as the first meal (breakfast) suppresses interstitial glucose elevations after the second meal (lunch). (A) Postprandial interstitial glucose level fluctuations after the first and second meals. (B) Maximum change in the interstitial glucose levels after the second meal. (C) Incremental area under the interstitial glucose curve (IAUC) after the second meal. Values are expressed as the mean ± SEM (*n* = 10). *p < 0.05, **p < 0.01, and ***p < 0.001. Statistical significance in (A) was assessed using the Friedman test, followed by Dunn's multiple‐comparison test versus white rice. Statistical analyses in (B) and (C) were conducted via one‐way repeated‐measures ANOVA, followed by Dunnett's post hoc test versus white rice.

Effects of the first meal on the interstitial glucose levels after the second meal are shown in Figure 5A and Table S5. Similar to that observed in Experiment 1, interstitial glucose levels after the consumption of fruit granola, baked oatmeal, and cooked oats as the first meal were lower than those after the consumption of white rice. Moreover, no notable increase in interstitial glucose levels was observed when breakfast was skipped.

In terms of interstitial glucose levels after the second meal, only the group consuming cooked oats as the first meal exhibited a suppressed postprandial glucose spike compared to the other groups.

As shown in Figure 5B and Table S6, maximum change in interstitial glucose levels following the second meal was low only when cooked oats were consumed during the first meal compared to white rice. Furthermore, IAUC after the second meal was lower in the cooked oat group than in the white rice group (Figure 5C; Table S6).

Our results suggest that cooked oats exert the second‐meal effect, efficiently attenuating the postprandial glucose responses to subsequent meals.

## 4 Discussion

In this study, two human intervention trials were performed to evaluate the effects of oat‐based foods on the postprandial blood glucose and insulin levels. In Experiment 1, we compared the postprandial glucose and insulin dynamics with various oat‐based foods and white rice. In Experiment 2, we investigated whether oat‐based foods exert the second‐meal effect, as assessed by the glycemic responses to a subsequent meal.

### 4.1 Acute Glycemic and Insulinemic Responses to Oat‐Based Foods: Mechanisms and Interpretation

Experiment 1 showed that oat‐based foods, such as fruit granola, baked oatmeal, and cooked oats, were associated with smaller fluctuations and slower increase in the postprandial blood glucose levels than white rice. Notably, both baked oatmeal and fruit granola exhibited low IAUC values and were classified as low‐GI foods. Additionally, analysis of insulin responses revealed that fruit granola and baked oatmeal enhanced the postprandial insulin secretion. The observed reduction in blood glucose levels after oat‐based food consumption is possibly due the presence of β‐glucan, a water‐soluble dietary fiber abundant in oats (Aoe 2016). The mechanisms by which β‐glucan suppresses postprandial blood glucose elevation possibly include its viscosity, resulting in delayed gastric emptying (Hossain et al. 2025; Wolever et al. 2020). β‐glucan also reduces the α‐glucosidase activity and glucose transport functions of glucose transporter 2 and sodium–glucose cotransporter 1 (Malunga et al. 2021), thereby inhibiting glucose absorption in the small intestine. β‐glucan promotes short‐chain fatty acid (SCFA) production in the large intestine (Guzowska et al. 2024; Mio, Iida‐Tanaka, et al. 2022; Mio, Ogawa, et al. 2022). SCFAs bind to the G‐protein‐coupled receptors (e.g., GPR41) on colonic L cells, stimulating the secretion of glucagon‐like peptide‐1 (GLP‐1), which promotes insulin secretion (Holst 2007). However, human studies using fermentable fibers such as inulin indicate that colonic fermentation–driven changes in circulating SCFAs and gut hormones generally emerge several hours after ingestion, rather than within the early 0–2 h postprandial window (Tarini and Wolever 2010). Furthermore, cereal‐based evening meals rich in indigestible carbohydrates improved glucose tolerance and altered GLP‐1 concentrations at a standardized breakfast meal the following morning (approximately 10–14 h after the evening meal), rather than within the first 2 h after intake (Nilsson et al. 2010). Therefore, the acute insulin responses observed over 0–120 min in the present trial are more likely to reflect small‐intestinal mechanisms, including delayed gastric emptying, reduced intestinal glucose transport, and direct incretin and insulinotropic effects of the co‐ingested nutrients, than SCFA‐mediated GLP‐1 secretion. The SCFA–GLP‐1 pathway may instead contribute to longer‐term improvements in glycemic control during sustained oat consumption, which was beyond the scope of this acute study.

### 4.2 Fiber Fractions, β‐Glucan Functionality, and Apparent Viscosity: Explaining the Lower Early Insulin Response to Cooked Oats

Notably, Table 2 shows that fruit granola containing the lowest β‐glucan levels among the tested oat‐based foods, was associated with lower blood glucose levels and strong insulin responses compared to the other oat‐based foods. With the fiber composition data, cooked oats provided the largest amounts of total dietary fiber (9.2 g), including both soluble fiber (5.4 g) and insoluble fiber (3.8 g), as well as the highest β‐glucan content (3.4 g), whereas fruit granola and baked oatmeal provided 5.7–6.3 g total fiber with 3.5–3.7 g soluble fiber and 2.2–2.6 g insoluble fiber. Despite this higher fiber load, cooked oats elicited a lower insulin response than fruit granola and baked oatmeal. Another plausible explanation is that the glycemic and insulinemic effects of oat fiber depend not only on the dose of β‐glucan but also on its molecular weight–dependent viscosity and extractability, which can influence small‐intestinal rheology and nutrient diffusion (Wolever et al. 2020). Although β‐glucan molecular weight could not be measured in this study, we conducted an additional functional assessment by measuring the apparent viscosity of the aqueous phase obtained after a standardized oral‐processing simulation (Table S7). Interestingly, the apparent viscosity values of the diluted supernatants were low (approximately 1–3.5 mPa·s) and did not correlate with β‐glucan dose; cooked oats showed a lower apparent viscosity than white rice, whereas fruit granola showed a modestly higher value. These results suggest that aqueous‐phase viscosity under the present in vitro conditions alone is insufficient to explain the lower early insulin response of cooked oats. Rather, differences in food matrix structure, hydration behavior, and the rate of starch digestibility/absorption—together with co‐ingested nutrients—may have contributed to the observed 0–120 min insulin dynamics in this acute setting. Accordingly, the lower insulin response to cooked oats may reflect a combination of factors (e.g., intact grain structure and moisture‐rich matrix leading to slower carbohydrate availability).

### 4.3 Contribution of Co‐Ingested Nutrients: Milk Protein and Dietary Fat

In contrast, fruit granola and baked oatmeal were consumed with milk and contained higher amounts of protein and fat than cooked oats and white rice. Rapidly digested dairy proteins, particularly whey, are known to have marked insulinotropic effects through aminoacidemia and stimulation of incretin hormones such as GIP and GLP‐1, and co‐ingestion of milk with cereal‐based foods can augment the insulinemic response while attenuating postprandial glycemia (Nilsson et al. 2004; Ostman et al. 2001). Dietary fat further modulates gastric emptying and incretin secretion, as shown in human studies where manipulation of fat digestion or fat load altered gastric emptying rates and GLP‐1/GIP responses to mixed meals (Feinle‐Bisset et al. 2005; Gentilcore et al. 2006; Pilichiewicz et al. 2003). Therefore, the greater insulin responses observed after fruit granola and baked oatmeal are likely to reflect the combined effects of oat‐derived fiber and these compositional differences rather than the action of β‐glucan alone.

### 4.4 Soluble Versus Insoluble Fiber: General Evidence and Relevance to the Present Findings

For instance, insoluble dietary fibers or soluble fibers inhibit glucose absorption in the intestine (Ge et al. 2024; He et al. 2023). Previous studies have reported functional distinctions between the two types of dietary fibers. A meta‐analysis found that soluble fiber improved insulin sensitivity, whereas insoluble fiber was more effective in lowering HbA1c levels (Lee et al. 2023). However, a recent systematic review noted that soluble fiber enhances insulin sensitivity in individuals with normal weight but does not consistently improve postprandial glucose levels, while in overweight individuals, it contributes to glycemic improvements. In contrast, the evidence linking insoluble fiber with either glycemic or insulinemic outcomes remains limited and inconclusive (Tsitsou et al. 2023). Notably, consuming a combination of soluble and insoluble dietary fibers alters the gut microbiota composition and increases SCFA production more than consuming either fiber type alone (Sasaki et al. 2020). Furthermore, the relative proportion of soluble‐to‐insoluble fibers in the diet affected the amount of SCFAs produced, with different ratios resulting in varying levels of SCFA production (Zhang et al. 2024). Nevertheless, in the present study, the soluble‐to‐insoluble fiber ratios of baked oatmeal and cooked oats were comparable, and cooked oats showed a lower (not higher) early insulin response despite the highest fiber and β‐glucan contents. This pattern suggests that fiber ratio alone is unlikely to account for the observed insulinemic differences within 0–120 min, and that co‐ingested milk protein and fat/protein content (as well as food structure/viscosity factors) may have played an important role in observed acute insulin dynamics under the present conditions. Plant‐based proteins in oats may also contribute to glycemic regulation. Soy protein intake increases the insulin‐like growth factor‐1 levels and improves glucose metabolism (Deibert et al. 2011), suggesting that plant‐derived proteins play a role in glycemic control. Furthermore, the potential influence of co‐consumed milk with fruit granola and baked oatmeal should be considered when interpreting the results. Milk possesses insulinotropic properties (Ostman et al. 2001; Sueda et al. 2009), which possibly contribute to postprandial blood glucose suppression. In this study, fruit granola and baked oatmeal were consumed with milk, whereas cooked oats and white rice were consumed without milk; thus, co‐ingested milk protein and the higher protein and fat contents of these meals should be regarded as important confounding factors when interpreting the insulinemic responses and the underlying mechanisms. β‐glucan possibly interacts with other nutrients to exert differential effects on blood glucose regulation and insulin secretion. However, we did not assess gastric emptying, amino acid profiles, or incretin hormone levels in the present trial; thus, the proposed mechanisms remain speculative. Future studies should clarify the mechanisms by which oat‐based foods exert glycemic control by examining not only blood glucose and insulin responses but also parameters such as glucose absorption rate, gastrointestinal transit time, and postprandial hormone responses.

### 4.5 Impact of Processing on β‐Glucan Functionality and Postprandial Responses

In addition to their nutritional composition, processing and cooking methods of oats possibly influence the postprandial blood glucose responses. Finely milled or crushed oat products, such as oat flour and instant oats, are more rapidly digested and absorbed than coarser oat products, such as steel‐cut and unprocessed whole oats, resulting in marked blood glucose level elevation (Wolever et al. 2019; Zhou et al. 2024). Furthermore, oats subjected to extensive processing, such as extrusion or roasting, elicit strong glycemic responses, possibly due to the breakdown of the native structural integrity during such treatments (Granfeldt et al. 2000). Molecular weight of oat β‐glucan is correlated with its viscosity and absorption characteristics in the small intestine. Refining and heat treatment reduce the molecular weight and viscosity of β‐glucan, potentially diminishing its physiological functionality (Wang and Ellis 2014). In this study, oat‐based foods were processed using different methods, which possibly contributed to the observed differences in glycemic responses and GI values. However, detailed physical and structural characteristics, such as particle size and molecular weight of β‐glucan, of each product were not assessed. Therefore, further investigation of the physiochemical properties of β‐glucan is necessary to elucidate its role in postprandial glycemic regulation.

### 4.6 Second‐Meal Effect Assessed by Continuous Glucose Monitoring System: Findings and Potential Mechanisms

In Experiment 2, we investigated the second‐meal effects of oat products using a continuous interstitial glucose monitoring system. Consuming cooked oats as the first meal was associated with a lower postprandial interstitial glucose response after the second meal under the present conditions, whereas fruit granola and baked oatmeal did not show a second‐meal effect under the present conditions. According to the nutrient analyses (Table 2), cooked oats provided the largest amount of total dietary fiber and oat‐derived β‐glucan among the tested breakfasts, despite all first meals being matched to 50 g of available carbohydrates. Similar second‐meal effects have been reported when cereal‐based meals rich in indigestible carbohydrates, such as barley kernels, were consumed at the previous meal, leading to improved glucose tolerance and altered GLP‐1 and free fatty acid profiles at the subsequent standardized meal (Johansson et al. 2013; Nilsson et al. 2008). These findings support that a higher load of slowly fermentable dietary fiber at the first meal, such as the larger β‐glucan dose provided by cooked oats in our study, may contribute to a more pronounced second‐meal effect. In addition, cooked oats retained a relatively intact grain structure and higher moisture content compared with the more extensively processed baked products. These factors may have resulted in greater viscosity in the small intestine and/or slower digestion and absorption, thereby leading to more pronounced effects on postprandial glycemic responses to the subsequent rice meal. While the present study does not disentangle the relative contributions of β‐glucan dose, fiber type, and food structure, the observed pattern suggests that cooked oats have properties that favor the second‐meal effect. Several mechanisms have been proposed to explain the second‐meal effect. For example, composition of the first meal possibly improves the glucose uptake efficiency and enhances the insulin sensitivity during the second meal (Lu et al. 2023; Waterman et al. 2024). Dietary fiber intake possibly also stimulates the secretion of incretin hormones, such as glucose‐dependent insulinotropic polypeptide and GLP‐1, which promote insulin secretion and influence glycemic control (Kim, Nanba, et al. 2020; Kuwahara et al. 2020). In contrast, elevated free fatty acid levels are associated with decreased insulin sensitivity (Roden et al. 1996). Skipping meals prolongs the fasting period, leading to increased free fatty acid levels and postprandial increase in glucose levels (Kuwahara et al. 2022; Xiao et al. 2022). Delayed gastric emptying by dietary fibers is another possible mechanism blunting glucose excursions after the second meal (Jenkins et al. 1982). Wolever et al. reported that the viscosity of oat‐derived β‐glucan affects the gastric emptying rate and postprandial glucose and insulin levels but does not influence the levels of intestinal hormones, such as GLP‐1, ghrelin, and peptide YY, suggesting that these hormones are not the primary mediators of the second‐meal effect (Wolever et al. 2020). Here, insulin and GLP‐1 levels were not measured in Experiment 2, as blood sampling was not performed. Therefore, the proposed mechanisms remain hypothetical in the context of this study. Future studies should incorporate hormonal analyses with continuous glucose monitoring to elucidate the mechanisms underlying the second‐meal effect. Furthermore, whether the processing or cooking of oat products are associated with the postprandial hormone levels warrants further investigation to provide deeper insights into the mechanisms by which oat‐based foods modulate glycemic regulation via delayed or attenuated interstitial glucose responses.

### 4.7 Study Limitations and Future Directions

This study has some limitations. First, numbers of participants analyzed in experiments 1 and 2 were relatively small (11 and 10, respectively), which reduced the statistical power compared with the a priori target sample size derived from our power calculation and limits the precision and generalizability of our findings. This study followed the guidelines established by the Japanese Association for the Study of Glycemic Index (Matsuoka et al. 2023); however, caution should be warranted when interpreting the results and generalizing them to a broader Japanese adult population. Additionally, all participants were healthy adults; therefore, the findings may not be applicable to individuals with impaired glucose tolerance, such as those with prediabetes, or to older adults, who may have different glycemic responses. In addition, the present study should be interpreted as an exploratory randomized crossover trial designed to compare relative responses under standardized conditions, rather than to estimate population‐level effects of oat consumption in Japanese adults. Future studies with larger and more diverse populations are needed to validate and expand upon these results.

Second, fasting blood samples were collected within a 1‐h window (8:00–9:00 a.m.) rather than at a single fixed time point. Although all measurements were conducted in the early morning after an overnight fast from 10:00 p.m., and each participant served as his or her own control with postprandial glycemic responses evaluated as incremental changes from the fasting value, minor variability in fasting glucose due to circadian rhythms in hormones such as cortisol and insulin cannot be completely excluded.

Third, the macronutrient composition and protein sources were not matched across the test meals. While the portion sizes were standardized so that each meal provided 50 g of available carbohydrates, fruit granola and baked oatmeal were consumed with milk and contained higher amounts of protein and fat than cooked oats and white rice. These compositional and matrix differences may confound the interpretation of insulinemic responses and make it difficult to attribute the observed effects solely to oat β‐glucan or dietary fiber. In addition, we did not measure β‐glucan molecular weight or directly quantify in vivo extractability/intestinal viscosity, which are key determinants of β‐glucan functionality. Although we assessed the apparent viscosity of diluted aqueous extracts under standardized in vitro conditions (Table S7), this measure may not fully represent the rheological environment in the gastrointestinal tract. Therefore, the contribution of β‐glucan physicochemical properties to the observed responses could not be fully evaluated.

Fourth, participant lifestyles and dietary habits possibly influenced the study outcomes. Although all participants were instructed to fast from the night before the test, individual variability in baseline eating patterns and lifestyle behaviors possibly affected the postprandial responses. Therefore, study designs should consider the habitual diets and daily routines of participants to obtain more ecologically valid results.

Fifth, this study only assessed short‐term effects by evaluating the postprandial interstitial glucose levels and insulin dynamics within a few hours of meal consumption. Long‐term effects of sustained oat consumption, such as changes in glycemic control and insulin sensitivity, were not examined, warranting further longitudinal studies to assess the chronic impact of oat‐based foods on metabolic health.

Finally, test meal quantities, particularly those in Experiment 2, possibly affected the findings. In this study, the second meal was standardized to 150 g of white rice (50 g carbohydrates) for consistent comparisons. This standardized meal composition was determined based on evidence from a previous short‐term human study (Fuse et al. 2020; Matsuoka et al. 2020). Moreover, the second meal consisted solely of white rice without accompanying protein, fat, vegetables, or other side dishes, in order to isolate the second‐meal effects on postprandial glycemia. However, this quantity possibly does not reflect the typical lunch portions in real‐life settings. Therefore, our findings may differ under more practical dietary conditions, necessitating caution when using these results for general dietary guidance.

Future studies should address these limitations via improved study designs, including increased sample sizes, evaluation of long‐term outcomes, incorporation of study protocols reflecting the real‐world dietary habits and lifestyles, and examination of diverse test meals, to provide robust and generalizable evidence of the potential health benefits of oat‐based foods. Despite these limitations, the crossover design and consistent within‐participant comparisons provide a robust basis for evaluating relative differences among oat‐based products under standardized conditions.

## 5 Conclusions

Our pilot study results suggest that some oat‐containing foods may help moderate postprandial glycemic responses under the present experimental conditions. Specifically, baked oatmeal and fruit granola were classified as low‐GI foods in this study, and cooked oats showed a second‐meal effect under the present conditions.

Therefore, consumption of some oat‐based foods possibly contributes to glycemic control in healthy Japanese adults. However, further studies are needed to determine whether similar second‐meal effects occur with other oat‐based products, different doses of β‐glucan, or more complex mixed meals.

## Author Contributions

**Yuma Matsumoto:** writing – original draft, formal analysis, data curation, investigation. **Hiroyuki Sasaki:** formal analysis, writing – original draft, data curation, investigation. **Katsuyuki Ishihara:** conceptualization, writing – review and editing, supervision. **Hirofumi Masutomi:** formal analysis, investigation, conceptualization, writing – review and editing. **Shigenobu Shibata:** conceptualization, writing – review and editing, supervision. **Kazuko Hirao:** writing – review and editing, conceptualization, supervision. **Akiko Furutani:** conceptualization, supervision, writing – review and editing, formal analysis, investigation.

## Funding

This work was funded by Calbee Inc.

## Ethics Statement

The study adhered to the ethical principles outlined in the Declaration of Helsinki and was approved by the Ethics Review Committee of Aikoku Gakuen Junior College. The trial was registered on the University Hospital Medical Information Network (UMIN Clinical Trials Registry ID: UMIN000048192).

## Consent

Written informed consent was obtained from all participants before study enrollment.

## Conflicts of Interest

H.S., Y.M., H.M., and K.I. are employees of Calbee Inc. This study was funded by Calbee Inc.

## Supporting information


**Table S1:** Ingredient lists of the oat‐based products used in this study.
**Table S2:** Mean blood glucose levels and standard errors at each time point after test meal consumption in Experiment 1.
**Table S3:** Values of glucose and insulin response in Experiment 1.
**Table S4:** Mean blood insulin levels and standard errors at each time point after test meal consumption in Experiment 1.
**Table S5:** Mean interstitial glucose levels at each time point after test meal consumption in Experiment 2.
**Table S6:** Values of glucose and insulin response in Experiment 2.
**Table S7:** Apparent viscosity of aqueous extracts after standardized oral‐processing simulation.

## Data Availability

The study data, codebook, and analysis code are available upon reasonable request from the corresponding author.
