# Supplementary material for: Acute and Second‐Meal Effects of Oat Products on Postprandial Glucose Responses in Healthy Japanese Adults: A Randomized Crossover Pilot Study
Source: Food Sci Nutr. 2026 May 24;14(5):e71791. doi: 10.1002/fsn3.71791 (PMC13240552; doi:10.1002/fsn3.71791)
Supplement: Supplementary file 1 — Table S1: Ingredient lists of the oat‐based products used in this study. Table S2: Mean blood glucose levels and standard errors at each time point after test meal consumption in Experiment 1. Table S3: Values of glucose and insulin response in Experiment 1. Table S4: Mean blood insulin levels and standard errors at each time point after test meal consumption in Experiment 1. Table S5: Mean interstitial glucose levels at each time point after test meal consumption in Experiment 2. Table S6: Values of glucose and insulin response in Experiment 2. Table S7: Apparent viscosity of aqueous extracts after standardized oral‐processing simulation. [file FSN3-14-e71791-s001.docx]

**Supplemental information**

**Supplemental method**

**Apparent viscosity measurement of aqueous extracts after standardized oral-processing simulation**

To obtain a standardized aqueous extract reflecting the apparent viscosity of each test meal after oral processing under controlled conditions, viscosity was measured using a rotational viscometer.

Each test meal (white rice, fruit granola, baked oatmeal, cooked oats) was weighed to 10 g (wet weight) into a sterile stomacher bag and then 30 mL of 37°C warm water was added to the bag. Test meals were homogenized using a stomacher (Promedia SH-ⅡM; ELMEX Co., Ltd., Tokyo, Japan) for 60 s to standardize bolus formation and disperse the food matrix, and the homogenate was kept in a 37°C water bath for 5 min to equilibrate temperature and hydration. Test meals were centrifuged at 1200 rpm for 5 min to separate insoluble particulates and the supernatant was collected. Apparent viscosity of the supernatant was measured at 37°C using a rotational viscometer (Brookfield DV1 VISCOMETER; spindle SC4-18, AMETEK Brookfield, MA, USA), with rotation speed fixed at 100 rpm. Measurements were continued for 5 min, and the value at 5 min was recorded as the apparent viscosity (mPa·s). Data are reported as mean ± SEM. Statistical comparisons versus white rice were conducted using one-way ANOVA with Dunnett’s post-hoc test (two-sided), with p < 0.05 considered significant.

Supplemental TABLE 1: Ingredient lists of the oat-based products used in this study

| Fruit granola | Baked oatmeal | Cooked oats |
| --- | --- | --- |
| Oats, dried fruit (papaya, raisins, apples, strawberries), rye flour, sugar, wheat flour, coconut, dextrin, vegetable oil, rice flour, corn flour, soluble dietary fiber, pumpkin seeds, almond powder, salt, wheat bran, brown rice flour, fructooligosaccharide syrup, lactose / glycerin, sodium citrate, acidulant, antioxidants (vitamin E, rosemary extract), modified starch, niacin, calcium pantothenate, vitamin A, vitamin B6, vitamin B1, folic acid, vitamin D, vitamin B12 | Oats, dextrin, vegetable oil, maltooligosaccharide, soluble dietary fiber, salt, lactose, almond milk, sodium ferrous citrate, antioxidants (vitamin C, vitamin E) | Oats |

Supplemental TABLE 2: Mean blood glucose levels and standard errors at each time point after test meal consumption in Experiment 1.

| Blood glucose  [mg/dL] | 0 min | 15 min | 30 min | 45 min | 60 min | 90 min | 120 min |
| --- | --- | --- | --- | --- | --- | --- | --- |
| White rice | 0.0 ± 0.0 | 13.3 ± 2.9 | 35.2 ± 4.1 | 42.6 ± 3.9 | 31.6 ± 6.0 | 26.4 ± 5.2 | 15.3 ± 5.4 |
| Fruit granola | 0.0 ± 0.0 | 11.9 ± 2.0 | 35.0 ± 3.6 | 18.5 ± 5.7^**^ | 5.0 ± 5.0^*^ | 6.0 ± 4.1^*^ | 1.7 ± 2.9 |
| Baked oatmeal | 0.0 ± 0.0 | 5.3 ± 1.3^*^ | 20.8 ± 3.0^**^ | 20.8 ± 4.4^**^ | 13.5 ± 3.9^*^ | 5.6 ± 3.6^*^ | 4.2 ± 3.6 |
| Cooked oats | 0.0 ± 0.0 | 9.3 ± 1.9 | 25.6 ± 2.5 | 25.4 ± 3.0 | 17.0 ± 4.3 | 12.6 ± 3.2 | 9.3 ± 2.5 |

Data are presented as mean ± SEM of the incremental blood glucose levels (mg/dL) at each time point (0, 15, 30, 45, 60, 90, and 120 minutes) for each test meal. Values at 0 min were set to 0 as baseline for all groups (n=11). Friedman test was applied, followed by Dunn’s multiple comparisons versus the White rice group, * p < 0.05, ** p < 0.01.

Supplemental TABLE 3. Values of glucose and insulin response in Experiment 1.

| Item | Blood glucose | | Blood insulin | |
| --- | --- | --- | --- | --- |
|  | Glucose max (mg/dL) | IAUC (mg*min/dL) | Insulin max (mU/L) | IAUC (mU*min/L) |
| White rice | 46.7 ± 3.7 | 3130.8 ± 419.1 | 19.8 ± 3.4 | 1257.6 ± 221.2 |
| Fruit granola | 36.5 ± 3.5^*^ | 1522.4 ± 261.4^**^ | 53.5 ± 12.4^*^ | 2446.1 ± 589.2^*^ |
| Baked oatmeal | 27.5 ± 3.3^***^ | 1367.4 ± 215.0^***^ | 38.1 ± 7.7^**^ | 1812.0 ± 344.6 |
| Cooked oats | 29.4 ± 2.8^**^ | 1817.0 ± 236.2^*^ | 21.6 ± 4.1 | 1227.1 ± 203.3 |

Data are presented as mean ± SEM (n=11). One-way repeated-measures ANOVA followed by Dunnett’s post hoc test for comparisons against the White rice group, * p < 0.05, ** p < 0.01, *** p < 0.001. IAUC, Incremental area under the curve.

Supplemental TABLE 4: Mean blood insulin levels and standard errors at each time point after test meal consumption in Experiment 1.

| Blood insulin  [mU/L] | 0 min | 15 min | 30 min | 45 min | 60 min | 90 min | 120 min |
| --- | --- | --- | --- | --- | --- | --- | --- |
| White rice | 0.0 ± 0.0 | 5.4 ± 1.4 | 13.0 ± 2.9 | 17.2 ± 4.0 | 12.7 ± 3.2 | 10.6 ± 2.1 | 7.8 ± 1.5 |
| Fruit granola | 0.0 ± 0.0 | 14.0 ± 2.8 | 50.5 ± 13.0^*^ | 35.0 ± 10.4 | 18.1 ± 4.5 | 14.9 ± 5.0 | 6.5 ± 3.0 |
| Baked oatmeal | 0.0 ± 0.0 | 7.1 ± 2.1 | 29.9 ± 8.9 | 30.0 ± 6.9^*^ | 16.4 ± 2.9 | 11.0 ± 2.2 | 6.8 ± 2.2 |
| Cooked oats | 0.0 ± 0.0 | 5.5 ± 1.0 | 19.3 ± 3.9 | 20.1 ± 4.3 | 11.8 ± 3.0 | 6.7 ± 1.1 | 5.7 ± 1.6 |

Data are presented as mean ± SEM of the incremental blood insulin levels (mU/L) at each time point (0, 15, 30, 45, 60, 90, and 120 minutes) for each test meal. Values at 0 min were set to 0 as baseline for all groups (n=11). Friedman test was applied, followed by Dunn’s multiple comparisons versus the White rice group, * p < 0.05

Supplemental TABLE 5: Mean interstitial glucose levels at each time point after test meal consumption in Experiment 2.

|  | First meal | | | | | | | | | | | | | | | | Second meal | | | | | | | | | | | | | |
| --- | --- | --- | --- | --- | --- | --- | --- | --- | --- | --- | --- | --- | --- | --- | --- | --- | --- | --- | --- | --- | --- | --- | --- | --- | --- | --- | --- | --- | --- | --- |
| Interstitial glucose  [mg/dL] | 0  h | 0.25  h | 0.5  h | 0.75  h | 1  h | 1.25  h | 1.5  h | 1.75  h | 2  h | 2.25  h | 2.5  h | 2.75  h | 3  h | 3.25  h | 3.5  h | 3.75  h | | 4  h | 4.25  h | 4.5  h | 4.75  h | 5  h | 5.25  h | 5.5  h | 5.75  h | 6  h | 6.25  h | 6.5  h | 6.75  h | 7  h |
| White rice | 0.0  ±  0.0 | 11.5  ±  2.5 | 34.5  ±  5.7 | 47.6  ±  7.6 | 45.7  ±  7.5 | 35.9  ±  6.1 | 23.8  ±  3.2 | 15.4  ±  3.1 | 10.6  ±  3.3 | 6.7  ±  2.7 | 5.1  ±  2.6 | 3.1  ±  2.2 | 0.1  ±  1.6 | -2.6  ±  1.4 | -4.0  ±  1.5 | -4.8  ±  2.0 | | 0.0  ±  0.0 | 9.3  ±  2.4 | 30.9  ±  4.5 | 50.7  ±  4.3 | 54.9  ±  5.0 | 47.1  ±  7.1 | 40.9  ±  9.2 | 35.9  ±  9.1 | 31.5  ±  7.5 | 24.0  ±  6.0 | 12.3  ±  4.2 | 4.2  ±  2.3 | 1.5  ±  2.7 |
| Fruit granola | 0.0  ±  0.0 | 13.8  ±  2.2 | 35.6  ±  4.2 | 40.1  ±  5.9 | 27.7  ±  5.3 | 16.7  ±  3.4 | 11.0  ±  1.5^*^ | 7.0  ±  1.9^*^ | 3.5  ±  2.2 | 2.3  ±  2.1 | 1.5  ±  2.1 | -0.1  ±  2.3 | -1.5  ±  2.6 | -2.1  ±  2.3 | -2.3  ±  2.1 | -3.3  ±  1.9 | | 0.0  ±  0.0 | 15.6  ±  3.2 | 41.5  ±  5.7 | 53.4  ±  5.8 | 51.2  ±  6.2 | 45.7  ±  5.5 | 38.2  ±  4.6 | 27.2  ±  3.8 | 17.1  ±  3.8 | 8.5  ±  3.6^*^ | 2.8  ±  2.9^*^ | 1.5  ±  3.1 | -0.2  ±  3.1 |
| Baked oatmeal | 0.0  ±  0.0 | 9.6  ±  1.8 | 28.0  ±  3.5 | 35.6  ±  5.4 | 27.3  ±  5.8 | 16.4  ±  4.6 | 9.8  ±  4.0 | 8.0  ±  3.8 | 9.0  ±  3.9 | 8.9  ±  3.8 | 7.5  ±  3.1 | 5.2  ±  2.3 | 2.8  ±  2.0 | 2.0  ±  1.7 | 2.6  ±  3.0 | 2.3  ±  3.4 | | 0.0  ±  0.0 | 16.6  ±  3.0 | 38.6  ±  3.7 | 49.5  ±  5.7 | 45.4  ±  6.3 | 34.8  ±  5.5 | 26.3  ±  5.2 | 20.4  ±  5.3 | 15.4  ±  4.8 | 7.9  ±  5.0 | 2.9  ±  5.6 | 0.4  ±  4.7 | -2.2  ±  3.5 |
| Cooked oats | 0.0  ±  0.0 | 11.2  ±  2.3 | 25.0  ±  2.8 | 24.8  ±  3.6 | 16.5  ±  3.5^*^ | 13.5  ±  2.7^*^ | 13.8  ±  2.6 | 11.5  ±  2.5 | 9.9  ±  2.1 | 9.2  ±  1.9 | 7.2  ±  2.8 | 3.4  ±  2.3 | 0.8  ±  1.6 | 1.1  ±  1.7 | 2.3  ±  1.8 | 2.7  ±  2.3 | | 0.0  ±  0.0 | 7.8  ±  3.1 | 20.5  ±  4.4 | 26.4  ±  4.0^**^ | 25.0  ±  5.6^***^ | 23.8  ±  5.0^*^ | 23.6  ±  3.5 | 23.3  ±  4.0 | 18.7  ±  3.5 | 8.8  ±  2.6 | 2.1  ±  3.2^*^ | 2.6  ±  4.1 | 2.1  ±  3.8 |
| Skipped breakfast | 0.0  ±  0.0 | -0.6  ±  1.0^**^ | -1.3  ±  1.3^***^ | -1.8  ±  1.6^***^ | -0.7  ±  1.2^***^ | 0.3  ±  1.2^**^ | 0.0  ±  1.5^***^ | -1.7  ±  1.3^**^ | -2.2  ±  1.4 | -1.1  ±  1.5 | -0.5  ±  1.5 | -1.0  ±  1.7 | -1.9  ±  1.9 | -2.1  ±  2.0 | -2.1  ±  3.1 | -2.1  ±  2.7 | | 0.0  ±  0.0 | 13.4  ±  2.1 | 39.8  ±  4.3 | 57.7  ±  4.3 | 58.6  ±  4.5 | 54.5  ±  5.2 | 45.9  ±  5.8 | 34.2  ±  5.3 | 24.5  ±  5.4 | 18.8  ±  5.1 | 14.8  ±  4.1 | 9.2  ±  3.1 | 3.7  ±  3.2 |

Data are presented as mean ± SEM of the incremental interstitial glucose levels (mg/dL) at each time point (0~3.75 hours for First meal, and 4~7 hours for Second meal) for each test meal. Values at 0 h and 4 h were set to 0 as baseline for all groups (n=10). Friedman test was applied, followed by Dunn’s multiple comparisons versus the White rice group, * p < 0.05, ** p < 0.01, *** p < 0.001

Supplemental TABLE 6. Values of glucose and insulin response in Experiment 2.

| Item | Glucose max (mg/dL) | IAUC (mg*h/dL) |
| --- | --- | --- |
| White rice | 60.4 ± 5.3 | 5216.3 ± 694.7 |
| Fruit granola | 58.9 ± 4.9 | 4696.5 ± 380.7 |
| Baked oatmeal | 53.5 ± 5.1 | 4139.4 ± 384.5 |
| Cooked oats | 37.1 ± 4.1^**^ | 2896.2 ± 258.1^*^ |
| Skipping breakfast | 65.3 ± 3.3 | 5620.7 ± 342.3 |

Data are presented as mean ± SEM. One-way repeated-measures ANOVA followed by Dunnett’s post hoc test for comparisons against the White rice group, * p < 0.05, ** p < 0.01. IAUC, Incremental area under the curve.

Supplemental TABLE 7. Apparent viscosity of aqueous extracts after standardized oral-processing simulation

| Test meal | Viscosity [mPa･s] | p-value (vs White rice) |
| --- | --- | --- |
| White rice | 2.41 ± 0.30 | - |
| Fruits granola | 3.45 ± 0.09 | 0.0038 |
| Baked oatmeal | 1.46 ± 0.03 | 0.0067 |
| Cooked oats | 1.34 ± 0.04 | 0.0033 |

Values are expressed as the mean ± standard error of the mean (SEM) (n=3). Apparent viscosity was measured at 37°C using a rotational viscometer (Brookfield DV1 VISCOMETER) with spindle SC4-18 at 100 rpm. p-values were calculated using one-way ANOVA with Dunnett’s post-hoc test.
